# Anti-FHL1 autoantibodies in adult patients with myositis: a longitudinal follow-up analysis

**DOI:** 10.1093/rheumatology/keae317

**Published:** 2024-06-04

**Authors:** Angeles S Galindo-Feria, Karin Lodin, Begum Horuluoglu, Sepehr Sarrafzadeh-Zargar, Edvard Wigren, Susanne Gräslund, Olof Danielsson, Marie Wahren-Herlenius, Maryam Dastmalchi, Ingrid E Lundberg, The SweMyoNet Consortium, Aladdin J Mohammad, Dag Leonard, Christopher Sjöwall, Thomas Husmark, Malin Ask, Silva Puksic, Theodoros Lappas, Balsam Hanna

**Affiliations:** Department of Medicine, Division of Rheumatology, Karolinska Institutet, Stockholm, Sweden; Center for Molecular Medicine, Division for Rheumatology, Department of Medicine, Solna, Karolinska Institutet, Stockholm, Sweden; Department of Gastro, Dermatology and Rheumatology, Theme Inflammation and Aging, Karolinska University Hospital, Stockholm, Sweden; Department of Medicine, Division of Rheumatology, Karolinska Institutet, Stockholm, Sweden; Center for Molecular Medicine, Division for Rheumatology, Department of Medicine, Solna, Karolinska Institutet, Stockholm, Sweden; Department of Gastro, Dermatology and Rheumatology, Theme Inflammation and Aging, Karolinska University Hospital, Stockholm, Sweden; Department of Medicine, Division of Rheumatology, Karolinska Institutet, Stockholm, Sweden; Center for Molecular Medicine, Division for Rheumatology, Department of Medicine, Solna, Karolinska Institutet, Stockholm, Sweden; Department of Medicine, Division of Rheumatology, Karolinska Institutet, Stockholm, Sweden; Center for Molecular Medicine, Division for Rheumatology, Department of Medicine, Solna, Karolinska Institutet, Stockholm, Sweden; Department of Medicine, Division of Rheumatology, Karolinska Institutet, Stockholm, Sweden; Department of Medicine, Division of Rheumatology, Structural Genomics Consortium, Karolinska Institutet, Stockholm, Sweden; Department of Medicine, Division of Rheumatology, Karolinska Institutet, Stockholm, Sweden; Department of Medicine, Division of Rheumatology, Structural Genomics Consortium, Karolinska Institutet, Stockholm, Sweden; Department of Biomedical and Clinical Sciences, Division of Neurology, Faculty of Medicine and Health Sciences, Linkoping University, Linkoping, Sweden; Department of Medicine, Division of Rheumatology, Karolinska Institutet, Stockholm, Sweden; Center for Molecular Medicine, Division for Rheumatology, Department of Medicine, Solna, Karolinska Institutet, Stockholm, Sweden; Department of Clinical Science, Broegelmanns Research Laboratory, University of Bergen, Bergen, Norway; Department of Medicine, Division of Rheumatology, Karolinska Institutet, Stockholm, Sweden; Center for Molecular Medicine, Division for Rheumatology, Department of Medicine, Solna, Karolinska Institutet, Stockholm, Sweden; Department of Gastro, Dermatology and Rheumatology, Theme Inflammation and Aging, Karolinska University Hospital, Stockholm, Sweden; Department of Medicine, Division of Rheumatology, Karolinska Institutet, Stockholm, Sweden; Center for Molecular Medicine, Division for Rheumatology, Department of Medicine, Solna, Karolinska Institutet, Stockholm, Sweden; Department of Gastro, Dermatology and Rheumatology, Theme Inflammation and Aging, Karolinska University Hospital, Stockholm, Sweden

**Keywords:** autoantibody, idiopathic inflammatory myopathy, myositis, FHL1

## Abstract

**Objectives:**

To determine prevalence and clinical associations of anti-Four-and-a-half-LIM-domain 1 (FHL1) autoantibodies in patients with idiopathic inflammatory myopathies (IIM) and to evaluate autoantibody levels over time.

**Methods:**

Sera at the time of diagnosis from patients with IIM (*n* = 449), autoimmune disease controls (DC, *n* = 130), neuromuscular diseases (NMDs, *n* = 16) and healthy controls (HC, *n* = 100) were analysed for anti-FHL1 autoantibodies by enzyme-linked immunosorbent assay (ELISA). Patients with IIM FHL1+ and FHL1− were included in a longitudinal analysis. Serum levels were correlated to disease activity.

**Results:**

Autoantibodies to FHL1 were more frequent in patients with IIM (122/449, 27%) compared with DC (autoimmune DC and NMD, 13/146, 9%, *P* < 0.001) and HC (3/100.3%, *P* < 0.001). Anti-FHL1 levels were higher in IIM [median (IQR)=0.62 (0.15–1.04)] in comparison with DC [0.22 (0.08–0.58)], HC [0.35 (0.23–0.47)] and NMD [0.48 (0.36–0.80)] *P* < 0.001. Anti-FHL1+ patients with IIM were younger at the time of diagnosis compared with the anti-FHL1− group (*P* = 0.05) and were seronegative for other autoantibodies in 25%.

In the first follow-up, anti-FHL1+ sample 20/33 (60%) positive at baseline had turned negative for anti-FHL1 autoantibodies. Anti-FHL1 autoantibodies rarely appeared after initiating treatment. Anti-FHL1 autoantibody levels correlated with CK (*r* = 0.62, *P*= 0.01), disease activity measured using the Myositis Disease Activity Assessment Tool (MYOACT) (*n* = 14, *P* = 0.004) and inversely with Manual Muscle Test-8 (*r* = −0.59, *P* = 0.02) at baseline.

**Conclusion:**

Anti-FHL1 autoantibodies were present in 27% of patients with IIM; of these, 25% were negative for other autoantibodies. Other autoimmune diseases had lower frequencies and levels. Anti-FHL1 levels often decreased with immunosuppressive treatment, correlated with disease activity measures at diagnosis and rarely appeared after start of treatment.

Rheumatology key messages

- A prevalence of 27% of anti-FHL1 autoantibodies in IIM was detected at baseline.
- Myositis-specific antibodies were not found in 25% of patients with anti-FHL1 autoantibodies.
- There was a correlation between FHL1 autoantibody levels and clinical outcomes at baseline.

## Introduction

The idiopathic inflammatory myopathies (IIM) are chronic, autoimmune disorders that comprise a heterogeneous group with distinct clinical phenotypes [1, 2]. These phenotypes can be identified based on clinical features, histopathological findings in muscle tissue and autoantibody profiles [3–5]. The umbrella term of IIM includes different disease subgroups including DM, PM, IBM, immune-mediated necrotizing myopathy (IMNM), antisynthetase syndrome (ASyS), clinically amyopathic dermatomyositis (CADM), overlap myositis and JDM [3, 6–8].

Myositis-associated autoantibodies (MAAs) or myositis-specific autoantibodies (MSAs) are detected in up to 80% of patients with IIM and are associated with specific clinical phenotypes [9, 10]. The relationship between clinical manifestations and autoantibody profiles has raised the question if MAA/MSA may have a role in the disease mechanisms of IIM. Of particular relevance in this context is the observation of changes in autoantibody levels in relation to disease activity and the disappearance of autoantibodies in patients who have gone into clinical remission [11].

One recent autoantigen identified in patients with IIM is a muscle-specific antigen, Four-and-a-half-LIM-domain 1 (FHL1), which is highly expressed in skeletal and heart muscle [12, 13]. FHL1 is involved in normal development, differentiation and interaction with cytoskeletal proteins in skeletal muscle. We have previously reported the presence of anti-FHL1 autoantibodies in patients with autoimmune diseases, with higher frequency in patients with IIM [12, 14]. Nevertheless, several questions remain unanswered.

A first question is whether anti-FHL1 autoantibodies are associated with a distinct clinical phenotype as the initial report in a European cross-sectional study identified anti-FHL1 autoantibodies to be associated with severe skeletal muscle involvement, but these findings could not be replicated in an independent Australian cohort [14]. Second, it is not clear if anti-FHL1 autoantibodies are a consequence of the inflammatory process in the muscle or if this autoantibody could be present already at the time of diagnosis and potentially have a role in the disease mechanisms. Third, do levels anti-FHL1 autoantibody vary with disease activity.

The aims of this study were to (1) determine the prevalence and clinical associations of anti-FHL1 autoantibodies, in a large cohort of patients with IIM; (2) to evaluate when anti-FHL1 autoantibodies are present in the course of the disease, and if autoantibody levels vary over time; and (3) to identify its presence in other autoimmune and neuromuscular diseases (NMDs). To accomplish these aims, we also validated the FHL1 enzyme-linked immunosorbent assay (ELISA).

## Patients and methods

### Patient population, sample collection and disease activity assessment

We included 449 adult patients with IIM identified from the Swedish myositis registry (SweMyoNet) with available serum samples. Clinical data and serum samples were collected between 1981 and 2019. The diagnosis of IIM and its subgroups was based on the 2017 EULAR/ACR classification criteria. ASyS was defined according to Connors’ criteria [15], and IMNM according to the 2017 ENMC criteria [16]. For seronegative IMNM, a muscle biopsy with dominant myofiber necrosis and a paucity of muscle inflammation was required [16]. Myositis not otherwise specified (MNOS) includes patients who presented with skeletal muscle inflammation but did not satisfy the subdiagnosis PM, DM, IBM, ASyS or IMNM. The diagnosis of mixed connective tissue disease (MCTD) was defined according the Alarcón-Segovia diagnostic criteria [17]. Diagnosis of interstitial lung disease (ILD) was based on the American Thoracic Society criteria [18].

For disease comparator, we included serum samples from patients with SS according to the 2002 American-European consensus Group (AECG) and 2016 ACR/EULAR classification criteria [19, 20]; SSc who fulfilled the 2013 ACR/EULAR classification criteria [21]; RA according to the ACR/EULAR 1987 or 2010 criteria [22, 23]; SLE according to the 1997 ACR criteria [24] and patients with NMDs. Furthermore, we included population-based controls without a rheumatic disorder (HC, *n* = 100).

From 2003 and onwards, information on disease activity and disease damage was evaluated prospectively at regular clinical visits following diagnosis of IIM and recorded in the SweMyoNet register. Disease activity was assessed as proposed by the International Myositis Assessment Collaborative Studies group (IMACS) including patient global disease activity (PGA), physician global disease activity (PhyGA), serum levels of creatine kinase (CK), manual muscle test-8 (MMT8), HAQ and extra-muscular disease activity using the Myositis Disease Activity Assessment Tool (MYOACT). For assessment of damage, the Myositis Damage Score (MYODAM) was used [25, 26]. Immunosuppressive treatment was decided by the treating physician.

### Ethics

This study was approved by the regional ethics committee, Stockholm (muscle biopsy and blood sample collection: 2005/792–31/4, 2006–712-32, 2011/1374–32, 2018/1198–32; disease and healthy control clinical information and blood samples collection from the SweMyoNet registry ethical permit: 2008/1457–31, 2012/736–32) and research subjects signed an informed consent according to the declaration of Helsinki.

### Autoantibody testing

Autoantibodies against Jo1, PL12, PL7, OJ, EJ, Zo, Mi2, MDA5, NXP2, TIF1γ, SAE1, SRP, HMGCR, PMScl, Ro52 and U1RNP were analysed using one or more of the following assays: (1) immunoprecipitation in collaboration with Dr Mimori, Japan, (2) Euroline myositis (panel 4 by Euroimmun, Lübeck, Germany) and/or (3) ELISA [27, 28]. Anti-HMGCR autoantibodies were kindly analysed by Dr Andrew Mammen, Muscle Disease Unit, National Institutes of Health, USA, by ELISA and confirmed by immunoprecipitation of *in vitro* transcribed and translated HMGCR protein (IP-IVTT).

### HLA typing

HLA typing for Class II alleles was performed for *HLA‐DRB1* alleles by sequence‐specific primer polymerase chain reaction assay (DR low‐resolution kit; Olerup SSP) [29].

### Muscle biopsy evaluation

Muscle biopsies underwent routine stains for inflammatory cells and were distinguished from non-inflammatory muscle conditions by routine use of histochemical and immunohistochemical techniques. Information from the biopsy reports was retrieved.

### Anti-FHL1 antibody ELISA

Serum samples, stored at −80°C until analyses, were selected from a time point close to diagnosis as baseline sample (index date). Samples for the longitudinal evaluation were selected from the anti-FHL1+ patients and from the anti-FHL1− patients according to the availability of consecutive sera in the biobank with a mean of 3 years (s.d. 2.65) between the index date or baseline (F1) and the follow-up samples (F2–F_n_).

Anti-FHL1 autoantibodies were analysed by indirect ELISA according to a protocol previously described [14]. The anti-FHL1 group was defined as the presence of anti-FHL1 autoantibody either at baseline or in at least one sample during the disease follow-up. Detailed information regarding the performance and validation of the ELISA testing is presented in the Supplementary Material, available at *Rheumatology* online.

### Statistical analyses

Descriptions of continuous variables are expressed as mean or median (s.d.) or interquartile range (IQR). Categorical variables are presented as frequencies and percentages. Differences between groups were analysed using the Student’s *t test* or Mann–Whitney *U* test, and Chi-squared or Fisher’s-exact test for categorical variables. Wilcoxon’s signed-rank test was used for comparison between longitudinal samples. The Kruskal–Wallis test was used for comparing the AU values of the different groups. Spearman correlation was used to correlate FHL1 levels and clinical outcomes. Pearson’s correlation was performed to analyse anti-FHL1 autoantibody levels and laboratory parameters. CK ratio was calculated using the CK-value divided by the upper limit of normal value. Longitudinal associations between anti-FHL1 autoantibody levels and clinical core set measures, disease activity and damage were tested by mixed effect regression models. Further details on the statistical analysis are presented in the Supplementary Material, available at *Rheumatology* online. GraphPad Prism 9.0 (GraphPad Software), IBM SPSS Statistic V.28 and STATA 16 (STATACorp, LP, TX, USA) were used for data management and statistical analyses. Two-tailed *P* values < 0.05 were considered statistically significant.

## Results

### Clinical and serological characteristics of patients with IIM, disease and healthy controls

The IIM cohort (*n* = 449), 282 (63%) women and 167 men, with a mean age (s.d.) at serum sampling of 56.8 (15.3) years. Disease subsets included DM (*n* = 129), PM (*n* = 194), ASyS (*n* = 102), IBM (*n* = 57), IMNM (*n* = 24), MCTD (*n* = 4) and MNOS (*n* = 39). The HC group consisted of 100 cases, 60/40 women/men with a mean age (s.d.) of 56 (15) years. The disease controls consisted of 146 cases, 127/19 women/men with a mean age (s.d.) of 51 (14) years and included SS (*n* = 100), SSc (*n* = 10), RA (*n* = 10), SLE (*n* = 10) and NMD (*n* = 16) with the following diagnoses: spinal muscle atrophy type 2 (*n* = 1), limb girdle muscle atrophy (*n* = 3), mitochondrial disease (*n* = 1), myotonic dystrophy type 1 (*n* = 3), congenital myasthenia (*n* = 1), and motor neuron disease (*n* = 1) and non-specified (*n* = 6). No significant differences were present in the IIM subtype or autoantibody profile (MSA and MAA) between anti-FHL1+ or anti-FHL1 negative groups. Ethnicity was recorded in 255/449 IIM patients; 249/255 (98%) were Caucasian and six African (2%) (Table 1).

**Table 1.** Characteristics at time of baseline serum sampling in patients with idiopathic inflammatory myopathies (IIM) included in the FHL1 ELISA analysis

	Anti-FHL1+ *n* = 122 (27%)	Anti-FHL1− *n* = 327 (73%)	*P* value
Female, *n* (%)	71 (58%)	211 (65%)	0.17
Age at serum sampling, mean, (s.d.)	54.5 (16.6)	57.7 (14.7)	0.05*
Age at diagnosis, mean (s.d.)	52 (15.9)	56 (16.9)	0.03*
FHL1 levels (AU) (median, IQR)	1.43 (1.23–2.13)	0.51 (0–0.76)	0.001**
Ethnicity, *n* (%)			
Caucasian	57/62 (92%)	186/193 (95%)	0.17
African	2/62 (3%)	5/193 (3%)	0.67
IIM subgroup, *n*(%)			
PM	25 (20%)	69 (21%)	0.88
DM	30 (25%)	99 (30%)	0.23
IBM	18 (15%)	39 (12%)	0.47
IMNM	7 (6%)	17 (5%)	0.81
MNOS	11 (10%)	28 (8%)	0.87
ASyS	30 (25%)	72 (22%)	0.61
MCTD	1 (1%)	3 (1%)	1.00
Antibody profile			
MSA, *n* (%)	115/122	309/327	
Jo1	25 (21%)	57 (17%)	0.43
Pl7	4 (3%)	9 (3%)	0.75
PL12	2 (2%)	6 (2%)	1.00
OJ	1 (1%)	1 (0.3%)	0.46
EJ	1 (1%)	2 (1%)	1.00
Zo	0 (0%)	1 (1%)	0.27
MDA5	3 (3%)	16 (5%)	0.30
HMGCR	3 (3%)	8 (3%)	1.00
SRP	4 (3%)	7 (2%)	0.49
TIF1γ	11 (9%)	32 (10%)	0.71
NXP2	2 (2%)	6 (2%)	0.70
SAE	3 (3%)	9 (3%)	1.00
Mi2	3 (3%)	9 (3%)	1.00
Number of MSA, *n*(%)			
Absent	54 (47%)	148 (48%)	0.86
MAA, *n*(%)	113/122	306/327	
Ro52	24 (21%)	63 (21%)	0.88
Ro60	15 (13%)	20 (7%)	0.03*
U1RNP	11 (10%)	26 (8%)	0.69
PMScl	16 (12%)	21 (7%)	0.02*
Ku	4 (4%)	9 (3%)	0.75
La	6 (5%)	2 (1%)	0.006**
MAA present^a^			
Absent	51 (54%)	192 (63%)	0.18
Negative MSA/MAA	28/113 (25%)	98/306 (32%)	0.29
Genetic, *n* (%)^a^	92/122	225/327	
HLA DRB1*01	24 (26%)	52 (23%)	0.83
HLA DRB1*03	56 (60%)	120 (53%)	0.30
HLA DRB1*04	19 (20%)	69 (30%)	0.23
HLA DRB1*07	8 (1%)	22 (1%)	0.76
HLA DRB1*08	9 (1%)	17 (1%)	0.43
HLA DRB1*09	1 (1%)	17 (1%)	0.19
HLA DRB1*10	1 (1%)	6 (3%)	0.67
HLA DRB1*11	15 (16%)	27 (12%)	0.37
HLA DRB1*12	0 (0%)	5 (2%)	0.32
HLA DRB1*13	18 (20%)	67 (30%)	0.12
HLA DRB1*14	5 (5%)	12 (5%)	1.00
HLA DRB1*15	25 (27%)	32 (14%)	0.02*
HLA DRB1*16	3 (3%)	9 (4%)	1.00
Clinical manifestations, *n*(%)			
Mechanic’s hands	15/88 (17%)	48/219 (22%)	0.33
Raynaud’s phenomenon	24/88 (27%)	61/219 (28%)	0.91
Arthritis	31/88 (35%)	71/218 (33%)	0.65
Calcinosis	4/88 (5%)	15/219 (7%)	0.44
Heliotrope rash	16/92 (17%)	65/240 (27%)	0.06
Gottron’s papules	24/92 (26%)	80/239 (34%)	0.19
Dysphagia	47/91 (52%)	124/238 (52%)	1.00
Malignancy	9/28 (32%)	44/94 (47%)	0.19
Interstitial lung disease	32/92 (35%)	81/242 (33%)	0.67
Muscle weakness	82/91 (90%)	224/240 (93%)	0.33
Skin ulcers	6/92 (7%)	19/239 (8%)	0.66
Laboratory evaluation, *n* (median, IQR)			
CK (microcat/l)	36/122 (25, 6.6–58.6)	79/324 (17.7, 6.7–77)	0.81
CRP (mg/ml)	11/122 (6, 4–65)	13/324 (10, 6–36)	0.64
ESR (mm)	29/122 (40, 17–60.5)	44/324 (18, 12–42.5)	0.03*
LD (microcat/l)	34/122 (13.7, 9.6–19.6)	53/324 (14.3, 8.8–21.4)	0.93
ALAT (microcat/l)	31/122 (1.2, 0.78–2.97)	54/324 (1.52, 0.97–2.6)	0.49
ASAT (microcat/l)	31/122 (1.34, 0.88–3.9)	54/324 (1.51, 0.82–2.5)	0.81

Results are expressed as *n* (%) if not otherwise specified.

a Not adjusted for multiple comparisons.

* *P* < 0.05.

** *P* < 0.01.

IIM: idiopathic inflammatory myopathies; MNOS: myositis not-otherwise-specified; IMNM: immune-mediated necrotizing myopathy; ASyS: anti-synthetase syndrome; MCTD: mixed connective tissue disease; MSA: myositis-specific autoantibodies; MAA: myositis-associated autoantibodies. ILD: interstitial lung disease. Laboratory evaluation of (*n*) available patients: CK: creatine kinase (reference value = < 3.5 microcat/l), CRP (reference value= < 3 mg/ml); ESR: erythrocyte sedimentation rate (reference value = < 20 mm); LD: lactic dehydrogenase (reference value = < 3.5 microcat/l); ALAT: alanine aminotransferase (reference value = < 0.76 microcat/l); ASAT: aspartate aminotransferase (reference value = <0.61 microcat/l).

**Figure 1.**
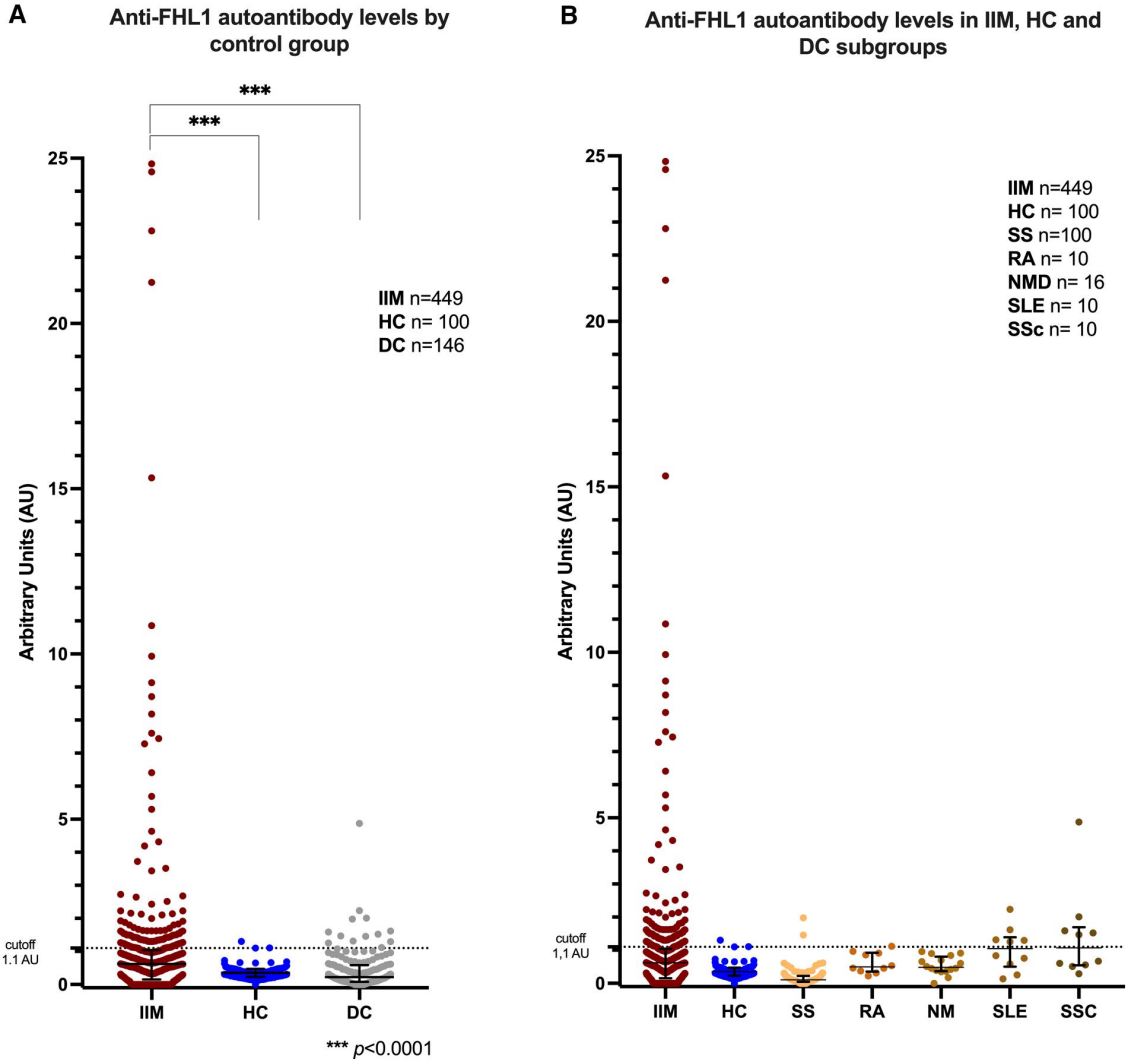
Anti-FHL1 autoantibodies in IIM and autoimmune disease controls. (A) Sera from patients with IIM (PM, DM, IBM, IMNM, ASyS, MCTD and MNOS; *n* = 449), HC (*n* = 100) and DC, including SS (*n* = 10), RA (*n* = 10), NMD (*n* = 16), SLE (*n* = 10) and SSc (*n* = 10), were analysed by ELISA using recombinant His-tagged FHL1. A cut-off value of 1.1 AU was calculated using a receiver operating characteristic (ROC) curve based on the HC. (B) Presence of anti-FHL1 autoantibody level by DC subgroup. Statistical analysis for (A–C) was performed using Kruskal–Wallis test with Dunn’s correction for multiple comparisons. Each point represents one individual, and horizontal lines indicate the median and interquartile range. Asterisk indicates a significant difference, *P values <0.0001. HC: healthy controls; IIM: idiopathic inflammatory myopathy; PM: polymyositis; DM: dermatomyositis; IBM: inclusion body myositis; IMNM: immune mediated necrotizing myopathy; ASyS: anti-synthetase syndrome; MCTD: mixed connective tissue disease; MNOS: myositis not-otherwise-specified; DC: disease controls; SS: Sjögren syndrome; RA: rheumatoid arthritis; NMD: neuromuscular diseases; SLE: systemic lupus erythematosus; SSc: systemic sclerosis

Autoantibodies to FHL1 were more frequent in patients with IIM at baseline (122/449, 27%) compared with DC (13/146, 9%, *P* < 0.001) and HC (3/100, 3%, *P* < 0.001) and not detected in the NMD group. Anti-FHL1 levels in baseline samples were higher in IIM [median (IQR)=0.62 (0.15–1.04)] compared with DC [0.22 (0.08–0.58)] and HC [0.35 (0.23–0.47)] *P* < 0.001 (Fig. 1A). In the DC group, anti-FHL1+ autoantibodies were present in SS (2/100, 2%), RA (1/100, 1%), SLE (5/10, 50%), and SSc (5/10, 50%) (Fig. 1B).

### Clinical and serological characteristics at baseline of patients with IIM

Clinical characteristics and autoantibody profiles of patients with IIM by anti-FHL1 autoantibody status at baseline are presented in Table 1. The anti-FHL1+ IIM group was younger at diagnosis and at the time of serum sampling compared with the anti-FHL1− IIM group (*P* = 0.03 and *P* = 0.05, respectively). No statistically significant differences in sex, ethnicity, IIM subgroup, or myositis-specific (MSA) autoantibodies were found when comparing patients with or without anti-FHL1 autoantibodies. Notably, 25% of anti-FHL1 autoantibody+ patients did not have any detectable MSA or MAA. HLA-DRB1*15 was more frequent in the anti-FHL1+ group (27% *vs* 14%, *P* = 0.02), though this difference was not statistically significant after adjusting for multiple comparisons. Anti-Ro60, anti-PMScl and anti-La autoantibodies were more frequent in the anti-FHL1+ group (13% *vs* 7%, *P* = 0.03; 12% *vs* 7%, *P* = 0.02 and 5% *vs* 1%, *P* = 0.006). ESR was significantly higher in the anti FHL1+ group (median 40 mm *vs* 18 mm, *P* = 0.03). Furthermore, there were significant correlations between anti-FHL1 autoantibody levels and LD (*r* = 0.58, *P* < 0.001), ASAT (*r* = 0.47, *P* = 0.006) and ALAT (*r *= 0.56, *P* ≤ 0.001).

### Anti-FHL1 autoantibody levels in the longitudinal follow-up of patients with IIM

**Figure 2.**
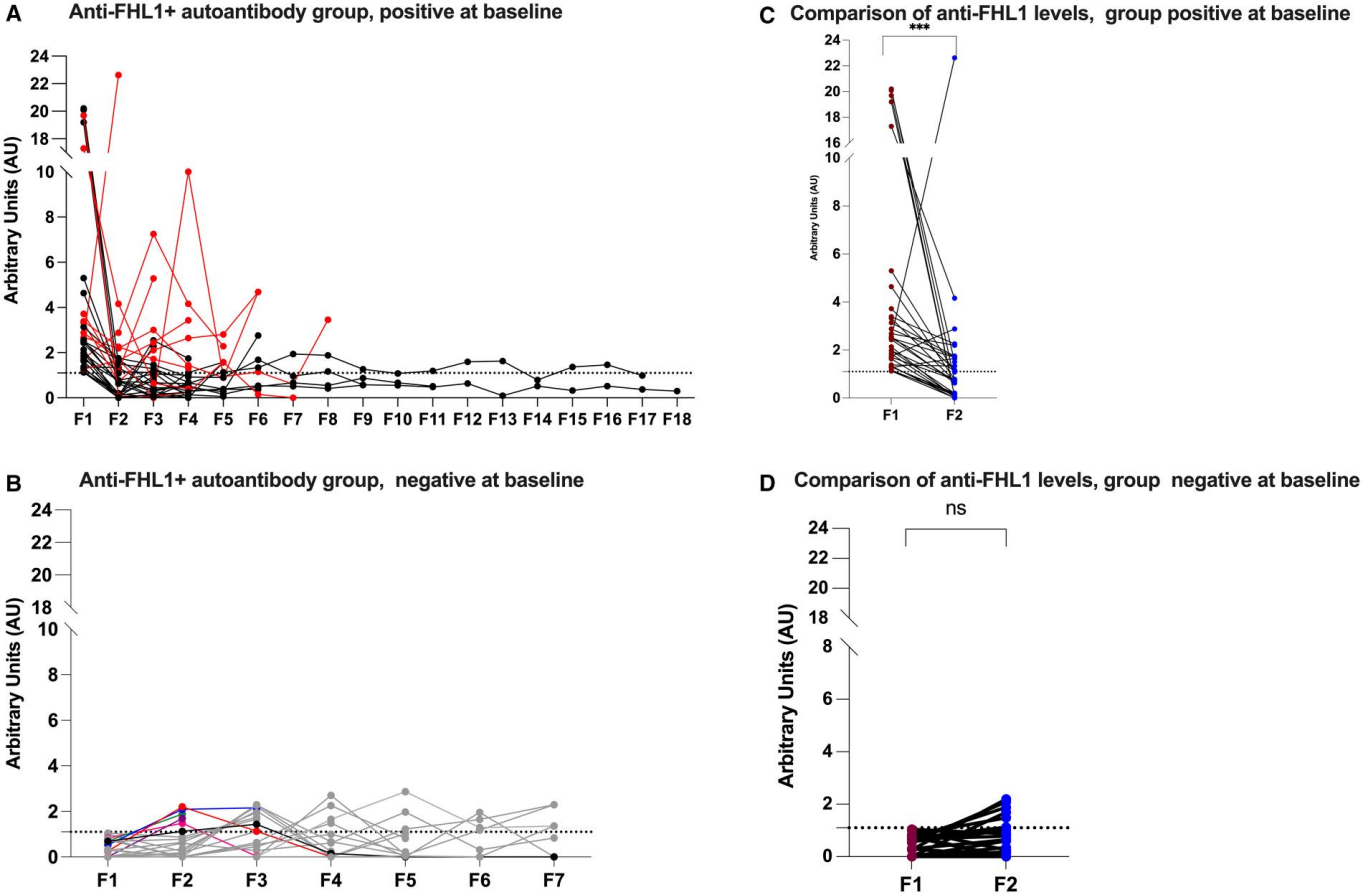
Longitudinal analysis of anti-FHL1 autoantibodies in IIM. Legend: Longitudinally collected sera from patients with IIM (anti-FHL1+ *n* = 57 and anti-FHL1− *n* = 30), where F1 represents the sample close to diagnosis (baseline) and F2-Fn the longitudinal samples. The anti-FHL1+ groups are represented according to serology status at baseline. The longitudinal data from the anti-FHL1− sera are not represented in this figure. (A) Group anti-FHL1+ at baseline (*n* = 33), where the red line and symbols indicate the serum samples with high anti-FHL1 autoantibody levels during follow-up. (B) Group anti-FHL1− at baseline (*n* = 24), where the coloured lines indicate individual samples that became positive in F2-F3. (C) Comparison of anti-FHL1 autoantibody levels at baseline and first follow-up in sera from patients belonging to the 2A. (D) Comparison of anti-FHL1 autoantibody levels at baseline and first follow-up in sera from patients belonging to the group 2B. Each point represents one serum sample and individual; horizontal lines indicate the longitudinal follow-up. Statistical analysis for (C, D) was performed using Wilcoxon matched-pairs signed rank test. Asterisk indicates a significant difference, ***P values <0.001, NS: non-significant

Longitudinal samples, when available, were obtained from patients with IIM from anti-FHL1+ (*n* = 57, median follow-up samples: 5) and anti-FHL1− (*n* = 30, median follow-up samples 4). Longitudinal clinical outcome measurements for a subgroup of 42/57 anti-FHL1+ and 25/30 FHL- IIM patients are presented in Table 2, Supplementary Fig. S1 and Table S1, available at *Rheumatology* online. The anti-FHL1+ group was defined by the presence of at least one positive test and further categorized into two subgroups: patients who were positive at the baseline sample (F1, *n* = 33, Fig. 2A), and those who transitioned to a positive status in at least one sample during follow-up (≥F2, *n* = 24) Fig. 2B).

**Table 2.** Clinical and serological characteristics of the 67 patients with idiopathic inflammatory myopathies (IIM) included in the longitudinal analyses defined by anti-FHL1 autoantibody status

	Anti-FHL1+ *n* = 42 (60%)	Anti-FHL1− *n* = 25 (40%)	*P* value
Women, *n* (%)	30 (71%)	15 (60%)	0.34
Age diagnosis, mean(s.d.)	51.3 (15.4)	52.2 (18.6)	0.84
Anti-FHL1 autoantibody levels (AU) (median, IQR)	3.68 (1.84–5.53)	0.21 (0.08–0.34)	0.005**
IIM subgroup, *n* (%)			
PM	10 (24%)	6 (24%)	0.55
DM	11 (26%)	8 (32%)	0.82
JDM	0 (0%)	1 (4%)	0.22
IBM	4 (10%)	1 (4%)	0.34
IMNM	2 (4%)	1 (4%)	0.53
MCTD	5 (12%)	2 (8%)	0.73
ASyS	10 (23%)	6 (24%)	1.00
Autoantibody profile			
MSA, *n* (%)			
Jo1	7/32 (10%)	5/25 (20%)	0.86
Pl7	1/32 (3%)	0/25 (0%)	0.37
PL12	2/32 (6%)	0/25 (0%)	0.20
OJ	0/32 (0%)	0/25 (0%)	N/A
EJ	0/32 (0%)	1/25 (4%)	0.25
MDA5	1/32 (3%)	2/25 (8%)	0.50
HMGCR	1/32 (3%)	1/25 (4%)	0.86
SRP	2/28 (7%)	0/25 (0%)	0.19
TIF1γ	2/21 (10%)	2/16 (13%)	0.77
NXP2	0/1 (0%)	0/2 (0%)	N/A
SAE	0/11 (0%)	0/5 (0%)	N/A
Mi2	3/31 (10%)	2/23 (9%)	0.90
Number of MSA, *n*(%)			
Absent	24/39 (62%)	12/25 (48%)	0.05*
MAA, *n*(%)			
Ro52	10/21 (48%)	2/14 (14%)	0.07
U1RNP	2/28 (7%)	4/24 (17%)	0.16
PMScl	9/32 (28%)	2/23 (9%)	0.11
Ku	1/31 (3%)	0/23 (0%)	0.41
La	0/17 (0%)	0/17 (0%)	
MAA present			
Absent	22/39 (56%)	19/25 (73%)	0.17
Clinical manifestations, *n* (%)			
Mechanic’s hands	9/33 (27%)	7/25 (28%)	0.95
Raynaud’s phenomenon	11/33 (33%)	8/25 (32%)	0.91
Arthritis	14/33 (42%)	13/25 (52%)	0.47
Calcinosis	5/33 (15%)	1/25 (4%)	0.17
Heliotrope rash	7/34 (21%)	7/25 (27%)	0.57
Gottron’s papules	12/34 (35%)	8/25 (31%)	0.71
Dysphagia	24/34 (71%)	18/25 (69%	0.91
Malignancy	1/13 (8%)	3/8 (38%)	0.09
Interstitial lung disease	8/34 (24%)	9/25 (35%)	0.35
Muscle weakness	32/33 (97%)	25/25 (100%)	0.37
Skin ulcers	1/34 (3%)	3/25 (12%)	0.19
Heart involvement	2/14 (14%)	0/25 (0%)	0.26
Histological features, *n* (%)			
Perifascicular atrophy	3/29 (10%)	4/19 (21%)	0.30
Perivascular inflammation	14/27 (52%)	7/19 (37%)	0.31
Endomysial infiltrate	13/30 (43%)	6/20 (30%)	0.34
MHC I upregulation	20/22 (91%)	8/14 (57%)	0.02*
Rimmed vacuoles	5/28 (18%)	2/20 (10%)	0.45

Results are expressed as *n* (%) if not otherwise specified.

* *P* < 0.05.

** *P* < 0.01.

N/A: not available.

Of the 33 patients who were positive in the baseline sample (F1), 20 patients (60%) had become negative for anti-FHL1 autoantibodies in the first tested follow-up sample (F2) after ∼36 months with immunosuppressive therapy.

Individuals who tested positive for FHL1 at baseline had higher CK (*P* = 0.05) and a trend towards lower MMT8 scores (73.5 *vs* 79, *P* = 0.08) compared with those initially negative but later seroconverted during follow-up. Despite higher disease activity in the FHL1+ group based on the MYOACT composite score at baseline, statistical significance was limited due to the small sample size. Notably, no significant differences were observed between the two groups in the first follow-up assessment (Supplementary Table S2, available at *Rheumatology* online).

The anti-FHL1 autoantibody levels in the first follow-up sample were significantly lower compared with the baseline samples (median AU 2.48 *vs* 0.76, *P* < 0.0001, Fig. 2C). There was no clinical difference in the comparison of disease activity between individuals transitioning to a negative status during the first follow-up (F1 *vs* F2, *n* = 20) and those who were still positive (*n* = 13) (Supplementary Table S3, available at *Rheumatology* online). However, there was a significant correlation between autoantibody levels and clinical outcomes reflected in the MYOACT index at baseline among anti-FHL1+ patients, whether they remained positive or became negative during the first follow-up (Supplementary Table S4, available at *Rheumatology* online).

Of the 24 patients who were anti-FHL1 negative in the baseline sample (F1), 6 patients (25%) had seroconverted to anti-FHL1 positive in the first follow-up sample (F2), with low levels, and the remaining 18 had seroconverted at various time points (Fig. 2B). Anti-FHL1 autoantibody levels in the group transitioning from negative to positive were significantly different between F1 and F3 (*P* = 0.045) (Fig. 2D). The mean duration for seroconversion from negative to positive during the follow-up was 5.75 years (±1.38, *P* = 0.045) in F1 compared with F3 (Supplementary Fig. S2, available at *Rheumatology* online).

The 30 seronegative cases remained seronegative through the observation period. They exhibited lower CK levels, less extra-muscular activity and higher MMT8 scores at baseline and during follow-up (3–5 years) compared with the anti-FHL1+ group (Table 3).

**Table 3.** Disease activity measures based on the available clinical data of patients in the longitudinal anti-FHL1 autoantibody cohort

		Anti-FHL1 positive patients		Anti-FHL1 negative patients	
	*n*	*n* = 44	*n*	*n* = 25	

Core set measures					*P* value
*index date*					
PGA, median (IQR)	12	49 (27.5; 69.5)	6	29 (16.0; 68)	0.27
PhyGA, median (IQR)	11	33 (10.0; 60.0)	6	10 (0.0; 17.0)	0.046*
CK, mean (s.d.)	10	4.22 (4.46)	5	0.59 (0.35)	0.070
MMT8, median (IQR)	10	73.5 (69.0; 78.0)	6	79 (76.0; 80.0)	0.085
HAQ^,^ median (IQR)	12	1.06 (0.44; 1.44)	6	0.63 (0.00; 1.00)	0.073
EM, median (IQR)	11	28 (10, 50)	6	5 (0; 34))	0.12
*3–5 years follow-up*		*n* = 32		*n* = 22	
PGA, median (IQR)	15	26 (13.0; 50; 0)	9	54 (47.0; 63.0)	0.069
PhyGA^,^ median (IQR)	12	5.5 (0.0; 20.5)	8	5.5 (0.0; 18.5)	0.94
CK, mean (s.d.)	13	1.88 (2.51)	7	0.89 (0.82)	0.33
MMT8, median (IQR)	14	74.0 (68.0; 79.0)	7	77.0 (68.0; 80.0)	0.29
HAQ^,^ median (IQR)	15	0.75 (0.50; 0.88)	9	1.00 (0.75; 1.50)	0.39
EM, median (IQR)	13	4 (0; 10)	9	0 (0; 7)	0.51

* *P* < 0.05.

Index date = first sample.

PGA: patient-completed global assessment, VAS 0–100; PhyGA: physician-completed global assessment, VAS 0–100; CK: creatinine kinase as ratio of ULN. MMT8: manual muscle test 8, 0–80; HAQ: health assessment questionnaire, 0–3; EM: extra-muscular disease activity, VAS 0–100.

### Clinical and histopathological characteristics at baseline for the longitudinal cohort of patients with IIM

For a subgroup of 42/57 anti-FHL1+ and 25/30 FHL-IIM patients, histopathological features in muscle biopsies were available from baseline and are presented in Table 2. The anti-FHL1+ group had a higher frequency of widespread MHC-1 upregulation on the sarcolemma of 91% (*n* = 20/22) *vs* 57% (*n* = 8/14) in the FHL1−, *P* = 0.02. There was no difference in distribution of IIM subgroups or other clinical or laboratory features at baseline between anti-FHL1 positive or negative patients.

### A comparison of disease activity in anti-FHL1 positive and negative patients

In a subgroup, repeated measurements of the six-item core set measures for evaluation of myositis disease activity were available at baseline for 12 anti-FHL1+ and 6 FHL-; and after 3–5 years for 15 anti-FHL1+ and 9 FHL1− patients with IIM (Table 3**)**. The anti-FHL1+ patients had significantly higher PhyGA (33 *vs* 10, *P* = 0.04), and a trend towards lower muscle strength at baseline as measured by MMT-8 in comparison with the anti-FHL1− group (73 *vs* 79, *P* = 0.08). They also had a trend towards higher scores of HAQ at baseline, indicative of a poorer physical function compared with the anti-FHL1− group (1.06 *vs* 0.63, *P* = 0.073) and higher CK (4.22 *vs* 0.59, *P* = 0.07). There were no statistically significant differences between the two groups regarding the remaining core set measures at baseline or at follow-up.

### Anti-FHL1 autoantibody levels and clinical outcome measures in a longitudinal observation

Associations between anti-FHL1 autoantibody levels and clinical outcome measures, available for up to 42 patients in the longitudinal part of the study, are presented in Table 4. At baseline, anti-FHL1 autoantibody levels correlated significantly with serum levels of CK (*n* = 15, *r* = 0.62, *P* = 0.01), the disease activity composite measure MYOACT (*n* = 14, *r* = 0.71, *P* = 0.004) and inversely with the MMT8 (*n* = 16, *r* = −0.59, *P* = 0.01). There was an inverse correlation between anti-FHL1 autoantibody levels and global damage at first follow-up (F2, *n* = 19, *r* = −0.44, *P* = 0.05). At the last recorded visit, there was a low correlation between anti-FHL1 autoantibody levels and cardiovascular damage (*n* = 42, *r* = 0.33, *P* = 0.03) and an inverse correlation with CK-levels (*n* = 42, *r* = −0.32, *P* = 0.04).

**Table 4.** Spearman’s correlations for the associations between anti-FHL1 autoantibody levels and disease activity measures and damage at baseline and follow-up in the longitudinal FHL1-cohort with available data

*baseline*	*n*	rho	*P* value
MMT8	16	−0.59	0.02*
CK	15	0.62	0.01**
MYOACT	14	0.71	0.004**
MDI extent	5	−0.06	0.92
MDI severity	5	−0.45	0.45
*measurement 2*			
MMT8	27	−0.02	0.92
CK	28	−0.01	0.97
MYOACT	15	0.37	0.18
MDI extent	16	0.07	0.81
MDI severity	17	−0.30	0.24
Global damage	19	−0.44	0.05*
Cardiovascular damage	20	−0.18	0.45
*last recorded visit*			
MMT8	41	0.22	0.16
CK	42	−0.32	0.04*
MYOACT	16	−0.26	0.34
MDI extent	36	0.07	0.69
MDI severity	35	−0.02	0.92
Global damage	42	−0.16	0.32
Cardiovascular damage	42	0.33	0.03*

MMT8: manual muscle test 8, 0–80; CK: creatinine kinase as ratio of upper limit normal; MYOACT (myositis disease activity assessment visual analogue scales) is the sum of the VAS scores for each of the items constitutional, cutaneous, skeletal, gastrointestinal, pulmonary and cardiac divided by the maximal possible score from those assessed items.

MDI extent of damage score is the sum of all scores for the 11 individual organ systems muscular, skeletal, cutaneous, gastrointestinal, pulmonary, cardiovascular, peripheral, vascular, endocrine, ocular, infection and malignancy divided by the total possible score of the assessed items.

MDI severity is the sum of the 10 cm visual analogue scale scores (0–100) for each of the 11 organ systems (muscular, skeletal, cutaneous, gastrointestinal, pulmonary, cardiovascular, peripheral vascular, endocrine, ocular, infection and malignancy) divided by the total possible score.

Global damage: VAS 0–100; cardiovascular damage: VAS 0–100.

* *P* < 0.05.

** *P* < 0.01.

### Associations between anti-FHL1 autoantibody levels and disease activity and damage

In 39 anti-FHL1+ patients with IIM with a total of 210 observations during follow-up, there were no statistically significant associations between anti-FHL1 autoantibody levels, core set measures, disease activity or damage in the mixed effect regression models including measurement from baseline and all available follow-ups. There was a significant association in the mixed effect regression models between higher levels of anti-FHL1 autoantibodies and younger age at onset of symptoms (b: −0.04, 95% CI −0.08; 0.00, *P* = 0.05), and younger age at diagnosis (b: −0.04, 95% CI −0.08; 0.00, *P* = 0.05). A graphical overview of two anti-FHL1 positive patients is provided in Supplementary Fig. S3 and S4, available at *Rheumatology* online.

## Discussion

In this study, we confirmed the presence of autoantibodies targeting the FHL1 protein in patients with IIM in 27% in the first available serum sample, but we also detected low levels of anti-FHL1 autoantibodies in patients with systemic sclerosis or SLE. In the longitudinal part of the study, we observed variations in the levels of anti-FHL1 autoantibodies in patients with IIM, and 60% of serum samples from the anti-FHL1 positive patients changed to a negative anti-FHL1 status in the first tested sera after ∼3 years. In addition, 25% of the initially anti-FHL1 negative patients exhibited a conversion to positivity in the first follow up samples, albeit with low levels of anti-FHL1 autoantibodies.

The frequency of anti-FHL1 autoantibodies in our cohort of patients with IIM is similar to the original report of anti-FHL1 autoantibodies but higher than in a more recently published study [14]. Our findings from the longitudinal follow-up, revealing a significant seroconversion rate to seronegative status for anti-FHL1 autoantibodies within the initial 3 years, provide a plausible explanation for the comparatively lower prevalence reported in the recently published cross-sectional study. In that study, the presence of anti-FHL1 autoantibodies was 14% with a median disease duration of 22.5 months [14]. There are several factors that could be associated with the conversion to negative status for anti-FHL1 autoantibody. One factor that could possibly influence a change from seropositive to seronegative is the immunosuppressive treatment as well as the response to treatment. However, this question could not be addressed in our study due to the limited available clinical data on disease activity in the follow-up cohort.

Importantly, our study supports the hypothesis that anti-FHL1 autoantibodies are present in the early phase of the IIM disease, arguing against that anti-FHL1 autoantibodies are a consequence of muscle inflammation or muscle damage in patients with IIM. In previous studies, we identified anti-FHL1 autoantibodies in patients with SSc in a low frequency. In this study, 5 out 10 SSc patients tested positive for the anti-FHL1 autoantibody with low-intermediate levels (AU 1.4–4.8). Future investigations will include an analysis of a larger scleroderma cohort. We also found low levels of anti-FHL1 autoantibodies in patients with other autoimmune diseases such as RA, SS and SLE but not in any of the individuals with NMD, indicating that this autoantibody can be present in other autoimmune diseases and is not specific for IIM but whether anti-FHL1 autoantibodies are associated with specific clinical manifestation in other autoimmune disorders needs to be investigated in larger cohorts.

One question that we wanted to address was if anti-FHL1 autoantibody levels correlate to disease activity or treatment response. A significant number of patients had become seronegative at the first time point selected for our follow up after 3 years, which might suggest effect of immunosuppressive treatment on autoantibody levels. Due to limited number of cases with persisting anti-FHL1 positive sera with corresponding disease activity data, it was not possible to assess if fluctuations in serum levels of anti-FHL1 followed fluctuations of disease activity.

The correlations between anti-FHL1 autoantibody levels and serum levels of muscle enzymes as well as the inverse correlation with the measure of muscle strength (MMT8) indicates an association between anti-FHL1 autoantibodies and disease activity in the skeletal muscle domain. Furthermore, there was a higher frequency of MHC-I upregulation in muscle fibers when compared with anti-FHL1 negative patients. While statistically significant, this finding was based on pathology reports in a small sample size.

We also tried to address whether anti-FHL1 autoantibody levels predicted the risk of damage in the follow-up part of the study, and our results suggest a possible linkage between anti-FHL1 autoantibody levels and cardiovascular damage at the last recorded visit. Importantly, the FHL1 protein is expressed not only in skeletal muscle but also in cardiac muscle. We were unable to ascertain the specific aetiology of cardiovascular damage, since this item relied on an overall score made by the treating physician using the MYODAM score. This observation warrants validation through a more extensive study (e.g. MRI, echocardiogram, etc.) for definitive confirmation.

Regarding the co-expression of other autoantibodies such as MAA/MSA, we found that almost 47% of anti-FHL1+ patients with IIM were seronegative for MSA and 54% for MAA, indicating that the anti-FHL1 autoantibody could be of added value in the diagnostic procedure in the seronegative IIM group. Furthermore, the high frequency of HLA-DRB*15 may indicate that this allele could be of relevance in this autoantibody subgroup, though we lacked statistical power to verify an association in this cohort.

There are some limitations in our study. Though this analysis was performed in a large multicentre cohort, some analyses were underpowered due to the low number of patients per subgroup or lack of clinical data. Some previously reported clinical associations with anti-FHL1 autoantibodies could not be replicated, such as dysphagia or vasculitis, which could have been overestimated in the original report on anti-FHL1 autoantibodies due to patient selection.

In conclusion, the present study provides additional information on anti-FHL1 autoantibody levels in adult patients with IIM. We report a 27% prevalence of anti-FHL1 autoantibodies close to time of diagnosis with a high frequency of seroconversion to anti-FHL1 negative sera during follow up, and with higher autoantibody levels in patients with IIM compared with disease and healthy controls. Furthermore, we found significant correlations between the serum levels of anti-FHL1 autoantibodies and muscle enzymes, as well with clinical measures of disease activity MMT8 and MYOACT, indicating a possible association to muscle involvement. Future studies *in vitro* and *in vivo* are required to understand the potential role of this autoantibody in the pathophysiology of autoimmune myopathies.

## Supplementary Material

keae317_Supplementary_Data

## Data Availability

Patient-level data, the statistical analysis and the dataset specifications are available for researchers who meet the criteria for access to confidential data. The local ethics committees will maintain the ethical restrictions of the data. Patient-level data will be anonymized, and study documents will be redacted to protect the privacy of the patients. Permission is required prior to access.
