# Supplementary material for: Anti-FHL1 autoantibodies in adult patients with myositis: a longitudinal follow-up analysis
Source: Rheumatology (Oxford). 2024 Jun 4;64(3):1482–92. doi: 10.1093/rheumatology/keae317 (PMC11879316; doi:10.1093/rheumatology/keae317)
Supplement: keae317_Supplementary_Data [file keae317_supplementary_data.zip › keae317_Supplementary_Data/rhe-23-2360-File005.docx]

**Supplementary Figure 1. Flow chart illustrating the patients with idiopathic inflammatory myopathies (IIM) included in this study for analyses of anti-FHL1 autoantibodies at baseline and in longitudinally collected samples**


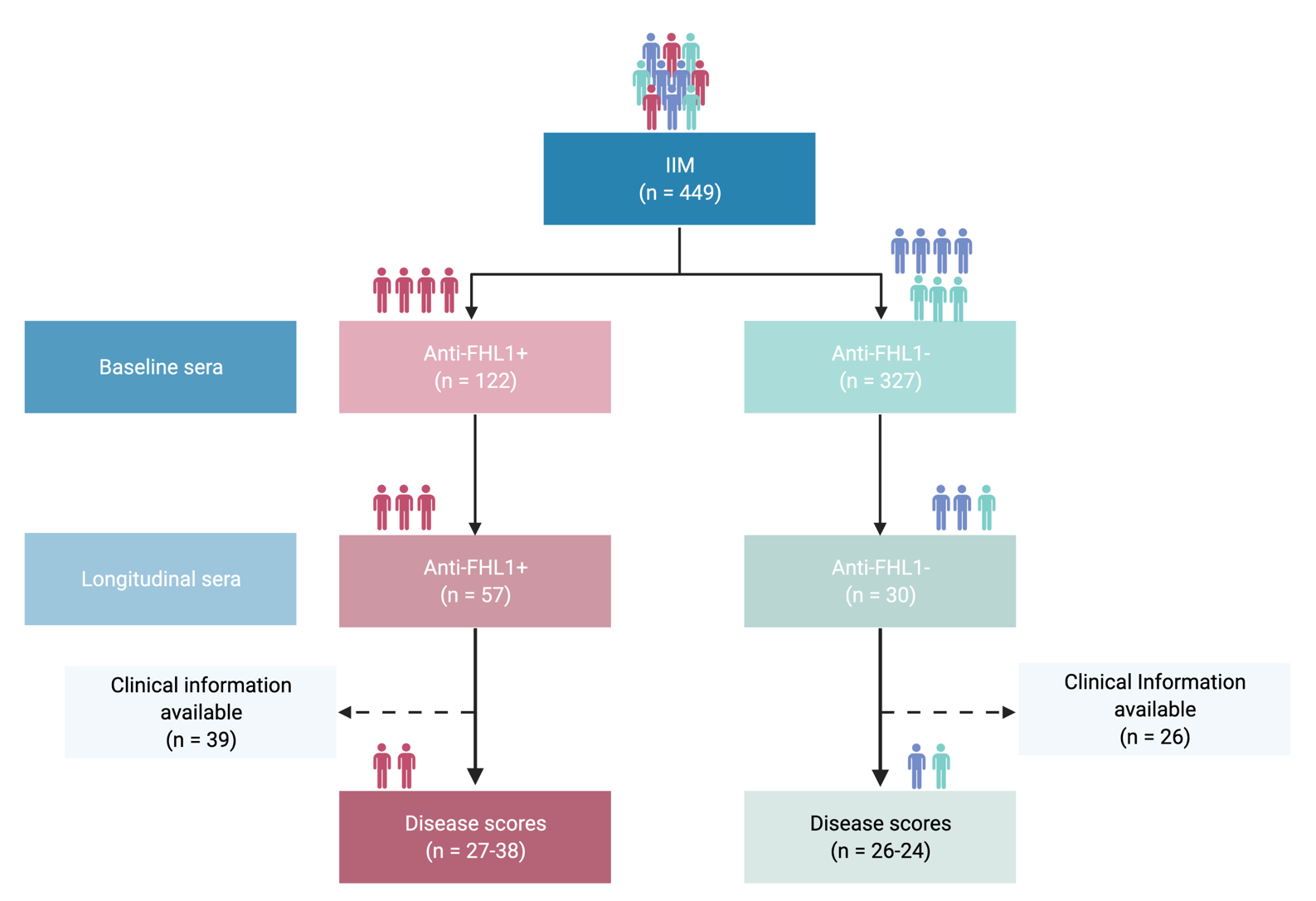


**Supplementary Figure 2:** **Anti-FHL1 negative patients at baseline that seroconverted during the longitudinal follow-up**

*p<0.05. The mean duration for seroconversion from negative to positive during the follow-up was 5.75 years (±1.38, p=0.045) in F1 compared to F3. Anti-FHL1 autoantibody levels presented in AU; dotted line represents the cut-off to positive anti-FHL1 status.


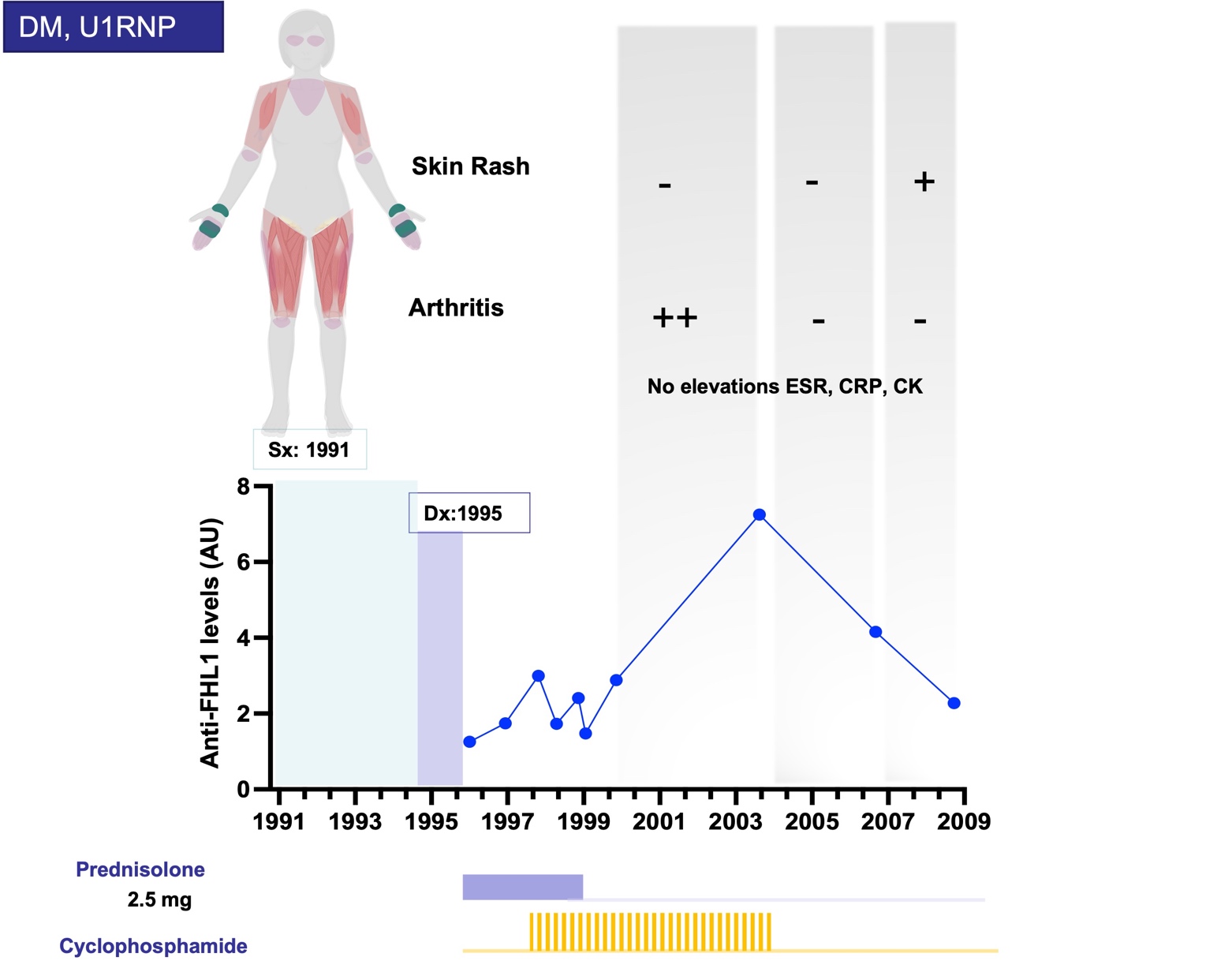


**Supplementary figure 3**: Longitudinal follow-up of a patient with FHL1+, dermatomyositis and U1RNP autoantibody. The FHL1 autoantibody levels in this patient correlated with disease activity such as arthritis which improved post-treatment, *aligning with a subsequent decrease in autoantibody levels*.


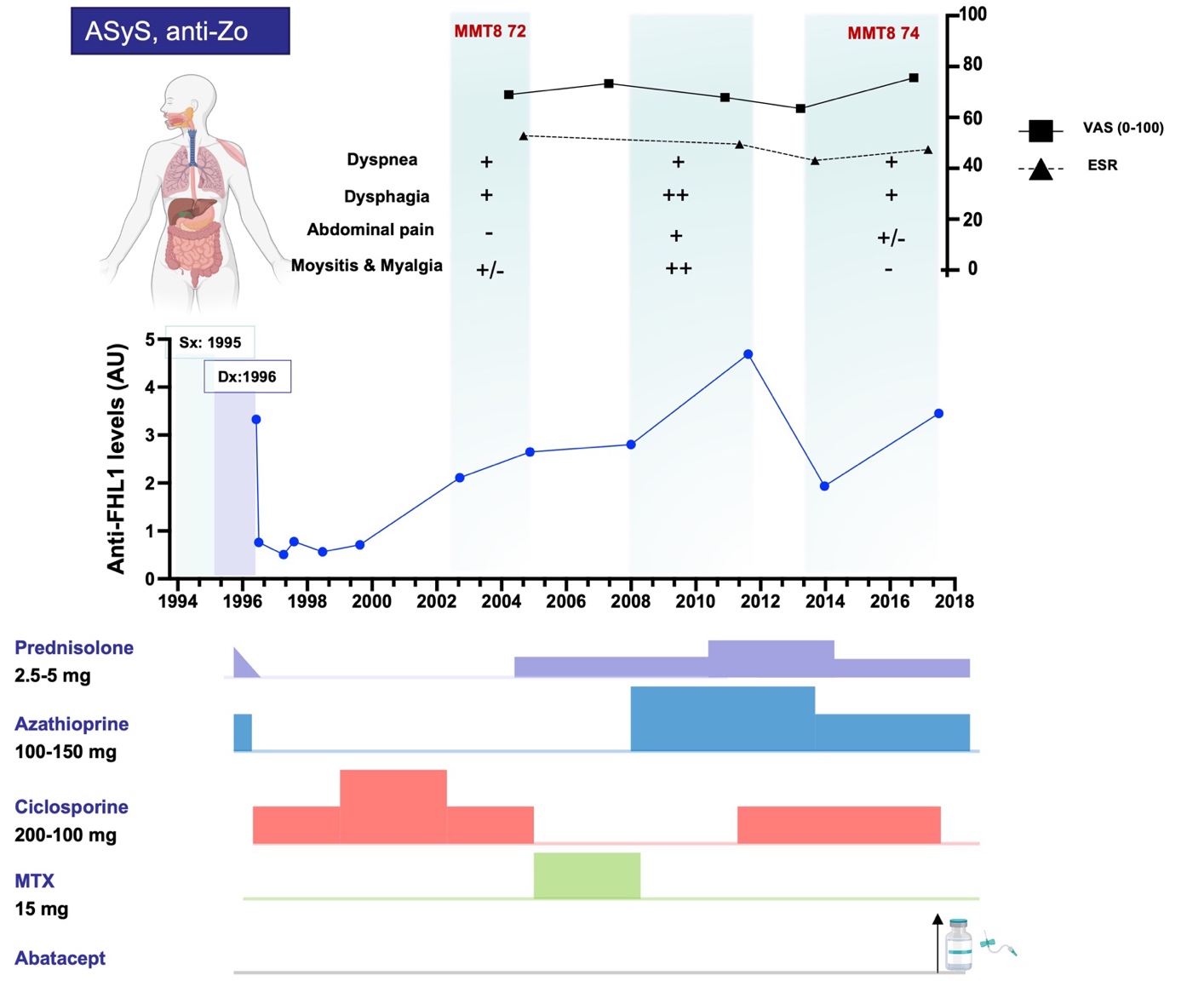


**Supplementary figure 4**: Longitudinal follow-up of a patient with FHL1+ and antisynthetase syndrome illustrating clinical manifestations, treatments, and fluctuations in the anti-FHL1 antibody levels. Notably, certain clinical features, including myositis and dysphagia, were more prevalent with increasing levels of autoantibodies.
