# Supplementary material for: Anti-FHL1 autoantibodies in adult patients with myositis: a longitudinal follow-up analysis
Source: Rheumatology (Oxford). 2024 Jun 4;64(3):1482–92. doi: 10.1093/rheumatology/keae317 (PMC11879316; doi:10.1093/rheumatology/keae317)
Supplement: keae317_Supplementary_Data [file keae317_supplementary_data.zip › keae317_Supplementary_Data/rhe-23-2360-File006.docx]

**Anti-FHL1 autoantibodies in adult patients with myositis: a longitudinal follow-up analysis**

**Supplement information for materials and methods**

Angeles S. Galindo-Feria^*^ ^1,2,3^, Karin Lodin^*^ ^1,2,3^, Begum Horuluoglu ^1,2^, Sepehr Sarrafzadeh-Zargar ^1,2^, Edvard Wigren ^1^,^4^, Susanne Gräslund ^1,4^, Olof Danielsson^5^, Marie Wahren-Herlenius ^1,2,6^ , Maryam Dastmalchi ^1,2,3^, Ingrid E. Lundberg ^1,2, 3^ and the SweMyoNet consortium

*Shared first authorship

^1^Division of Rheumatology, Department of Medicine, Solna, Karolinska Institutet, Stockholm, Sweden

^2^Center for Molecular Medicine, Karolinska Institutet, Karolinska University Hospital, Solna, Stockholm, Sweden

^3^Department of Gastro, Dermatology and Rheumatology, Karolinska University Hospital. Stockholm, Sweden

^4^Structural Genomics Consortium, Division of Rheumatology, Department of Medicine Solna, Karolinska Institutet, Stockholm, Sweden

^5^ Division of Neurology, Department of Biomedical and Clinical Sciences, Faculty of Medicine and Health Sciences, Linkoping University, Linkoping, Sweden

^6^ Broegelmann Research Laboratory, Department of Clinical Science, University of Bergen, Norway

***Characteristics of the cohort***

Patients with IIM in this cohort were regularly monitored with blood samples, including those negative for anti-FHL1 autoantibodies at baseline. All patients, irrespective of their anti-FHL1 status, underwent follow-up at the myositis clinic with clinical and serological analyses at baseline, after 3-6 months, and thereafter annually. Comparators were matched by sex and timing of blood sample in patients being either anti-FHL1 positive or anti-FHL1 negative at baseline to control for potential selection bias. There was no difference in frequency of blood sampling and anti-FHL1 analyses between those who were anti-FHL1 positive and anti-FHL1 negative at baseline

***Statistical analysis***

Mixed effect regression models: The overall longitudinal associations between anti-FHL1 autoantibody levels and clinical core set measures, disease activity and damage were tested by mixed effect regression models. All models were adjusted for sex. Furthermore, we investigated if treatment moderated the association between anti-FHL1 levels and clinical outcome measures by including an interaction term between treatment and anti-FHL1 titer in the mixed effect regression model.

Due to the non-normal properties of the included variables, the p-values in all mixed effect regression models were estimated by bootstrap with 2000 repetitions

***Anti-FHL1 antibody ELISA***

Anti-FHL1 autoantibodies were analyzed by indirect ELISA according to a protocol previously described (1). The OD values were transformed to Arbitrary Units (AU) by interpolation of a sigmoidal 4PL log_10_(x) standard curve consisting of 0.05 to 10 AU, where 1 AU=1:500 dilution of a standard serum sample that was used in all ELISA plates. The cutoff was calculated using a receiver operating characteristic (ROC) curve, including the HC. A cut-off of 1.1 AU units was considered positive, with a sensitivity of 23.65% (IQR 20.3%-27.3%), specificity of 97.5% (IQR 91.34-99-56%), likelihood ratio (LR) of 9.45 and area under the curve (AUC) of 0.74, p<0.0001. All samples were tested in duplicates and the analyses were performed in samples with an intra-assay coefficient of variability (CV) < 10% and inter-assay CV<15% (2).

1. **ELISA validation**

To confirm that the reactivity in the FHL1 ELISA was directed to FHL1 protein and not towards any contaminant, we compared the reactivity between FHL1 recombinant protein produced in E.coli with FHL1 protein produced in HEK cells or yeast.

The correlation between autoantibodies targeting the E.coli- and HEK-FHL1 proteins was good (r=0.79, p<0.001). The HEK-FHL1 ELISA had a sensitivity 58%, specificity 72%, and a likelihood ratio (LR) of 5.17. The comparison between E.coli and HEK-FHL1 protein demonstrated a similar specificity between these two proteins, but higher sensitivity and LR in the E.Coli FHL1 (sensitivity 67%, specificity 90%, LR 7.3). In addition we compared the reactivity between FHL1 protein produced in E.coli with FHL1 protein produced in yeast. The correlation between antibody levels targeting FHL1 protein from E.coli and yeast-FHL1 was excellent (r=0.96, p<0.0001) with a sensitivity of 57%, specificity 88% and LR 4.5.

We tested the reactivity of IIM and control samples using recombinant full-length human His-tagged FHL1 protein produced from *E.coli* and from HEK-293 cells, and evaluated the correlation between them. We included IIM FHL1+ (n=12), FHL1- (n=8), NMD (n=10) and HC (n=15) that were paired and tested simultaneously for all the conditions. Additionally, we compared the reactivity of IIM and control samples using recombinant FHL1 protein produced from yeast with our FHL1 from *E.Coli.* We included IIM FHL1+ (n=6), FHL1- (n=6), NMD (n=7) and HC (n=9) that were tested simultaneously in all conditions.

**Supplementary table 1. FHL1 levels at each measurement**

| Measurement | n | Mean | SD | Range |
| --- | --- | --- | --- | --- |
| F1 | 65 | 2.47 | 5.03 | 0;20.2 |
| F2 | 64 | 0.97 | 2.89 | 0;22.6 |
| F3 | 54 | 0.81 | 1.22 | 0;7.3 |
| F4 | 47 | 0.93 | 1.61 | 0;10 |
| F5 | 31 | 0.72 | 0.86 | 0;2.9 |
| F6 | 16 | 1.24 | 1.56 | 0;4.7 |
| F7 | 11 | 0.79 | 0.80 | 0;2.3 |
| F8 | 5 | 1.41 | 1.34 | 0;3.5 |
| F9 | 3 | 0.90 | 0.35 | 0.5;1.3 |
| F10 | 3 | 0.77 | 0.28 | 0.6;1.1 |
| F11 | 3 | 0.73 | 0.40 | 0.5;1.2 |
| F12 | 2 | 1.11 | 0.68 | 0.6;1.6 |
| F13 | 2 | 0.86 | 1.1 | 0.1;1.6 |
| F14 | 2 | 0.66 | 0.20 | 0.5;0.8 |
| F15 | 2 | 0.84 | 0.74 | 0.3;1.4 |
| F16 | 2 | 1.00 | 0.67 | 0.5;1.4 |
| F17 | 2 | 0.68 | 0.43 | 0.4;1.0 |
| F18 | 1 | 0.30 | . | . |

**Supplementary Table 2. Comparison between clinical outcomes at baseline and first follow-up stratified for FHL1 status at baseline**

|  |  | FHL1pos at baseline |  | FHL1neg at baseline | p-value |
| --- | --- | --- | --- | --- | --- |
| *Baseline* | n |  | n |  |  |
| MMT8 | 10 | 73.50 (69.00;78.00) | 7 | 79.00 (76.00;80.00) | 0.085 |
| CK | 10 | 2.56 (0.83;7.14) | 6 | 0.56 (0.31;0.66) | 0.051 |
| MYOACT | 10 | 0.10 (0.04;0.20) | 4 | 0.06 (0.02;0.10) | 0.320 |
|  |  |  |  |  |  |
| *First follow-up* |  |  |  |  |  |
| MMT8 | 15 | 76.00 (72.00;79.00) | 8 | 78.50 (68.50;80.00) | 0.600 |
| CK | 14 | 0.73 (0.37;1.24) | 9 | 0.69 (0.31;1.11) | 0.750 |
| MYOACT | 5 | 0.04 (0.03;0.07) | 6 | 0.03 (0.02;0.04) | 0.360 |

Data is presented as median and interquartile range (IQR). *p<0.05, **p<0.01, ***p<0.001. Mann-Whitney U-test for continuous data.

**Supplementary Table 3. Comparison between clinical outcomes stratified for FHL1 positivity during follow-ups at baseline and first follow-up**

|  | Turned negative | Persisting positive | p-value |
| --- | --- | --- | --- |
| *Baseline* | n=20 | n=13 |  |
| MMT8 | 76.5 (70.5;78) | 77 (75;78) | 0.77 |
| CK | 2.91 (0.86:11) | 0.64 (0.46;3.99) | 0.089 |
| MYOACT | 0.10 (0.07;0.18) | 0.10(0;0.20) | 0.74 |
|  |  |  |  |
| *First follow-up* | n=20 | n=13 |  |
| MMT8 | 75 (72;77) | 75 (71.5;79) | 0.81 |
| CK | 0.37 (0.29;0.69) | 0.49 (0.29;1.36) | 0.50 |
| MYOACT | 0.04 (0.03;0.07) | 0.08 (0.04;0.09) | 0.29 |

Data is presented as median and interquartile range (IQR). *p<0.05. Mann-Whitney U-test for continuous data

MMT8: Manual muscle test 8, 0-80; CK: Creatinine kinase as ratio of upper limit normal; MYOACT (myositis disease activity assessment visual analogue scales) is the sum of the VAS scores for each of the items constitutional, cutaneous, skeletal, gastrointestinal, pulmonary and cardiac divided by the maximal possible score from those assessed items.

**Supplementary Table 4. Correlation between FHL-1 antibody levels and clinical measures in patients divided according to seroconversion at baseline and in the first follow-up sample**

| *Baseline* | n | Turned anti-FHL1 negative (n=20) | n | Persisted anti-FHL1 positive (n=13) |
| --- | --- | --- | --- | --- |
| MMT8 | 8 | 0.379 | 5 | 0.088 |
| CK | 7 | 0.284 | 4 | 0.453 |
| MYOACT | 6 | 0.906* | 2 | 1.000* |
|  |  |  |  |  |
| *First follow-up* |  |  |  |  |
| MMT8 | 9 | 0.028 | 8 | 0.015 |
| CK | 9 | -0.055 | 8 | -0.052 |
| MYOACT | 3 | -0.999 | 4 | 0.393 |

*p<0.05. MMT8: Manual muscle test 8, 0-80; CK: Creatinine kinase as ratio of upper limit normal; MYOACT (myositis disease activity assessment visual analogue scales) is the sum of the VAS scores for each of the items constitutional, cutaneous, skeletal, gastrointestinal, pulmonary and cardiac divided by the maximal possible score from those assessed items.

**REFERENCES**

1. Galindo-Feria AS, Horuluoglu B, Day J, Fernandes-Cerqueira C, Wigren E, Graslund S, et al. Autoantibodies against Four-and-a-Half-LIM Domain 1 (FHL1) in Inflammatory Myopathies: Results from an Australian Single-Center Cohort. Rheumatology. 2022.

2. Liu X. Classification accuracy and cut point selection. Stat Med. 2012;31(23):2676-86.
